# Effect of wax separation on macro‐ and micro‐elements, phenolic compounds, pesticide residues, and toxic elements in propolis

**DOI:** 10.1002/fsn3.3866

**Published:** 2023-12-06

**Authors:** Eylul Evran, Serap Durakli‐Velioglu, Hasan Murat Velioglu, Ismail Hakki Boyaci

**Affiliations:** ^1^ Faculty of Engineering, Department of Food Engineering Hacettepe University Ankara Türkiye; ^2^ Faculty of Agriculture, Department of Food Engineering Tekirdag Namık Kemal University Tekirdağ Türkiye; ^3^ Faculty of Agriculture, Department of Agricultural Biotechnology Tekirdag Namık Kemal University Tekirdağ Türkiye

**Keywords:** macro‐elements, micro‐elements, pesticide residues, phenolic compounds, propolis, wax separation

## Abstract

Propolis, a natural product with many biological activities, is a resinous material produced by honeybees. It contains not only valuable components but also some possible contaminants in varying amounts. Hence, this study aimed to examine how the process step of wax separation affects certain elements, pesticide residues, and phenolic compounds in propolis. Total phenolics, elements, and some pesticide residues were analyzed in the crude propolis (CP samples), wax portion (W samples), and remaining propolis fraction (PF samples) after wax separation. Total phenolics of the CP samples were determined in the range of 31.90–45.00 mg GAE g^−1^ sample, while those of the PF samples were in the range of 54.97–162.09 mg GAE g^−1^ sample. Loss/reduction values by means of wax separation for phenolics were calculated as 10.88% and 17.89%, respectively. Pb contents of all PF samples were low (0.232–1.520 mg kg^−1^), but it was also noteworthy that nearly 40% or even more of Cr, As, Cd, and Pb were removed by wax separation. Removal of significant amounts of carbendazim (38.09%–67.35%), metalaxyl (81.57%–72.67%), tebuconazole (65.99%–78.36%), and propargite (88.46%–83.05%) was also achieved. Wax separation enables the removal of toxic substances from crude propolis without causing huge losses in phenolic compounds.

## Graphical abstract

Propolis, a natural product, contains valuable components along with potential contaminants. The separation of the wax portion allows the removal of significant amounts of some toxic metals, such as Cr, Pb, As, and Cd, and pesticides, including carbendazim, metalaxyl, tebuconazole, and propargite, without causing huge losses in phenolics. Wax separation could serve as a valuable process step for the reduction/removal of toxic contaminants in crude propolis.

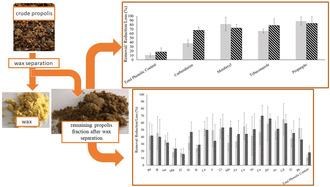

## 1 INTRODUCTION

Propolis, widely used as a traditional medicinal product for centuries, is a resinous hive product produced by honeybees (*Apis mellifera* L.) for the purposes of construction, adaptation, and protection in the hive. The material collected by honeybees from the leaves, buds, and exudates of trees and plants is partially digested by the β‐glycosidase in the bees’ saliva and then mixed with beeswax in order to form propolis (Bankova et al., 2000; Banskota et al., 2001; Daleprane & Abdalla, 2013; Ristivojević et al., 2015). The constituents of propolis make it an important product with a variety of biological activities, e.g. antioxidant, antibacterial, antiviral, anti‐fungal, and anti‐inflammatory properties (Bayram et al., 2020; Burdock, 1998).

The chemical profile of propolis is greatly affected by many factors, such as the species of bees, geographical origin, resources of plants, climate, collecting seasons, and production methods used (Kieliszek et al., 2023; Papotti et al., 2012; Ristivojević et al., 2015). In general, propolis contains resin and vegetable balsam (50%), wax (30%), essential and aromatic oils (10%), pollen (5%), and other substances (5%) such as amino acids, vitamins, and minerals (Huang et al., 2014). It can be regarded as a complex material, including more than 300 compounds that can be grouped as free phenolic acids, esters of these acids, flavonoids including flavones, flavanones, flavonols, and dihydroflavonols, chalcones and dihydrochalcones, terpenoids, and others (Ristivojević et al., 2015). As well as these phytochemicals, it is also a source of macro‐ and micro‐elements (Cvek et al., 2008). Researchers suggest that the synergistic action of these complex constituents results in the biological actions of propolis (Amoros et al., 1992; Bueno‐Silva et al., 2013; Cvek et al., 2008). Because of the wide range of biological activities of propolis, there is an increasing interest in products containing propolis, in parallel with the increasing consumer interest in natural functional products. Over the past five decades, scientific studies on propolis have also increased due to its pharmacological activity and its ability to prevent and treat many diseases (Bankova et al., 2019). There are many studies dealing with the use of propolis in various products in the food and cosmetic industries and as folk medicine. Crude propolis or extracts of it can be used in various products such as candies, drops, chewing gums, syrups, fresh juices, sprays, soaps, and toothpaste (Apaydin & Gümüş, 2018; Burdock, 1998; Ippolito et al., 2018; Juliano et al., 2007; Liao et al., 2021; Mohammadzadeh et al., 2007; Victorino et al., 2009).

Although there are quite a number of studies dealing with the health effects of propolis and its use in various products, there is limited research on the constituents of certain propolis fractions and the contaminants in them. In addition to the presence of contaminants in propolis from beekeeping practices, contaminants from agricultural activities (i.e. pesticides) and/or pollutants of the atmosphere and plants (i.e. toxic metals) can also be detected both in raw propolis and in its products (Blažková et al., 2022; Cvek et al., 2008; Gonzalez‐Martin et al., 2018). The monitoring of contaminants such as pesticide residues within propolis has gained increasing significance due to the rising utilization of propolis in recent years, driven by its perceived health benefits (Blažková et al., 2022). The study by Gonzalez‐Martin et al. (2018) reported that some of the processed propolis products contained different amounts of pesticides and toxic metals. Cvek et al. (2008) reported various levels of decrease in the toxic metal contents in the ethanolic extracts compared to their respective raw samples. The harvesting methods, pre‐processes, and/or extraction procedures used could also be influential in the concentrations of valuable constituents such as phenolic compounds and essential elements, as well as some contaminants in the propolis extracts (Bayram et al., 2020; Contieri et al., 2022; Cvek et al., 2008; Sales et al., 2006; Soós et al., 2019).

The processing of raw propolis includes limited steps. After the honey is extracted, beekeepers typically collect propolis by scraping it from various parts of the hive (Stawiarz & Dyduch, 2014). Propolis is usually found together with beeswax as a mixture; hence, the mixture of crude propolis and wax is shipped to the processor. It is reported that if the crude propolis is very waxy, it is washed with hot water to remove the extrinsic wax, and the remaining propolis is then air‐dried. If very little extrinsic wax is found, it goes immediately to the second step. In the extraction step, propolis is dissolved in 95% ethyl alcohol. This step and the final filtration enable the removal of the remaining beeswax as well as any foreign materials (Burdock, 1998). However, in the realm of propolis processing, it is a relatively uncommon practice to incorporate a dedicated step aimed at separating waxy constituents (Blažková et al., 2022; Catchpole et al., 2004).

The beeswax is of significant importance to the honey bee colony, as it mediates the acquisition of nestmate recognition cues in honey bees and captures toxins (Svečnjak et al., 2019). It is also reported to be the most contaminated hive compartment regarding the quantities of pesticides detected (Calatayud‐Vernich et al., 2018). Wax is also an important constituent in crude propolis. It affects the balsam percentage. The high percentage of balsam means the propolis contains a low percentage of wax and insoluble matter (Popova et al., 2007). Then the propolis with a higher wax content has a lower content of biologically active components. This suggested the separation of all wax from the crude propolis before the extraction step in order to increase the relative portion of active components in the propolis. On the other hand, the wax proportion could be a good solvent and/or carrier for some possible contaminants, and that is why the separation of wax from propolis is of great importance as it can affect the accumulation of contaminants. In most processes, there is usually no designated step for separating waxy materials, and to the best of our knowledge, there is no study about the effect of wax separation on the constituents of propolis, including contaminants. Thus, this study aimed to investigate the effects of the wax separation step on the components of propolis with the perspective of detecting valuable constituents as well as some contaminants such as toxic metals and pesticide residues.

## 2 MATERIALS AND METHODS

### 2.1 Reagents and solutions

The standards used (*p*‐coumaric acid, 3,4 dihydroxybenzoic acid, quercetin, caffeic acid, *t*‐ferulic acid, chlorogenic acid, gallic acid, epicathechin, pyracathechol, sinapic acid, vanilic acid, syringic acid, and phlorizin) and Folin–Ciocalteau reagent and acetonitrile were from Sigma–Aldrich (St. Louis, MO, USA). Pesticide standards were obtained as a mixture of reference components (CPAchem Ltd, Bogomilovo, Bulgaria). All reagents and standards used were HPLC grade, and purified water from a Milli Q system (Merck KGaA, Darmstadt, Germany) was used throughout the experiments.

### 2.2 Preparation of samples

Three different propolis samples with different wax portions were gathered as the products of the same season from different beekeepers located in different regions of Tekirdağ, Turkey. The samples from different beekeepers were used in this study to consider a possible range of variability in the wax content of the crude propolis samples. These samples were not subjected to a wax separation process and were used directly in the study. The wax was separated following the procedure below.

The samples from the same beekeeper were broken into small pieces and mixed, and three different master samples were obtained. One‐third of the samples were kept to represent the crude propolis samples (CP samples), and the remaining portion of the crude propolis samples was heated with continuous stirring at 80°C in a water bath for 1–2 h until the wax was clearly separated. The wax portion was taken, and the separation procedure was repeated twice until there was no wax phase separated. All of the portions were put into a freezer at −18°C. The frozen samples were taken and ground using a laboratory‐type grinder, and the samples were obtained in powder form. The samples were kept at −18°C until used. The samples were coded as CP samples (crude propolis), W samples (wax portion), and PF samples (propolis fraction after the wax separation). Hence, three groups of samples were used in the study, i.e. CP1, W1, and PF1; CP2, W2, and PF2; CP3, W3, and PF3. PF4 and CP4 samples were obtained from a non‐agricultural place to be used in recovery studies.

### 2.3 Extraction of phenolic compounds

The extraction step was carried out as described by Oruç et al. (2017) with slight modifications. Briefly, 1 g of propolis sample was extracted with 20 mL of 70% EtOH at ambient temperature for 1 h using a multi‐vortex (Multi Reax, Heidolph, Germany), and then the sample was ultrasonicated for 15 min (Wise Clean, DAIHAN Scientific, Korea). The propolis extract was filtered using Whatman filter paper (No: 1), and the filtrates were concentrated using a rotary evaporator at 40°C at 200 rpm. The dried extract was dissolved in 2 mL of absolute methanol and kept at 4°C until used. Appropriate dilutions were made, and the diluted extracts were used for the determination of phenolic compounds by LC–MS/MS and also for the determination of total phenolics (TP) content (Oruç et al., 2017).

### 2.4 Spectrophotometric detection of total phenolic (TP) content

TP analysis was performed using the Folin–Ciocalteu method (Singleton & Rossi, 1965). The extracts were diluted 250‐fold in order to be used in the TP assay. The experiment was conducted according to the microscale protocol (Matić et al., 2017) as follows: 20 μL of sample extract (or standard solution) in a glass tube was mixed with 1580 μL of distilled water, and then 100 μL of Folin–Ciocalteu reagent was added and mixed using a vortex (Reax top, Heidolph, Germany). 300 μL of Na_2_CO_3_ (200 gL^−1^) solution was added and mixed again. The tubes were incubated at 40°C for 30 min in a water bath (WiseBath, DAIHAN Scientific, Korea). The absorbance of the green‐blue complex was read at 765 nm against the blank solution using a UV–Vis spectrophotometer (UV‐2600, Shimadzu, Japan). A stock solution of gallic acid (1000 mgL^−1^) was used for the preparation of standard solutions having concentrations of 10, 25, 50, 100, 250, and 500 mgL^−1^. The calibration curve was generated using the absorbance values of the standard solutions, and this calibration curve was used for the calculation of the results. The results were expressed as mg gallic acid equivalents (GAE) per g sample.

### 2.5 Determination of some phenolics by LC–MS/MS

The extract was filtered and added to a glass vial. The sample was injected into the liquid chromatography coupled with tandem mass spectrometry (3200 QTRAP, AB Sciex LLC, MA, USA) system for analysis. An Agilent Poroshell 120 SB‐C8 LC column (3.0 × 100 mm, 2.7 μm) was used. Mobile phase A was a 0.2% aqueous formic acid solution, and Mobile phase B was a 0.2% formic acid solution in acetonitrile. The gradient program was as follows: Mobile phase A: 100% for 1.0 min. Mobile phase A: 100% for 30 s, Mobile phase A: 50% and Mobile phase B: 50% for 40 s, Mobile phase A: 20% and Mobile phase B: 80% for 2 min, 50 s, Mobile phase A: 50% and Mobile phase B: 50% for 2 min, and Mobile phase A: 100% for 1.0 min. The flow rate was 0.3 mL min^−1^. The column oven temperature was set at 40°C. Table S1 shows the LC–MS/MS method acquisition parameters of the analysis. The retention times (RT), ion transitions, declustering potential (DP), entrance potential (EP), and collision energy (CE) for the phenolics were given. Two multiple‐reaction‐monitoring (MRM) transitions were used. The first quantitation transition (MRM1) corresponds to the highest‐intensity ion transition, and the molecular weight of the compound was verified. The second transition corresponds to the confirmation transition (MRM2), and the fragment ions were scanned for mass determination (Jabot et al., 2015). The gas flow rate was 60 PSIG at a temperature of 500°C. Methanol was used for the preparation of the stock‐standard solutions of the phenolic compounds. The calibration curves were generated using five data points (0.1, 0.25, 0.5, 1, 2 mg kg^−1^).

### 2.6 Determination of mineral contents of propolis samples by inductively coupled plasma mass spectrometry (ICP‐MS)

Analysis of 20 elements (Be, B, Na, Mg, Al, Si, K, Ca, V, Cr, Mn, Fe, Co, Ni, Cu, Zn, As, Cd, Ti, and Pb) was performed using an Agilent 7700X ICP‐MS (Agilent, CA, USA). 0.5 g of homogenized sample was transferred into a Teflon vessel, and 5 mL of nitric acid and 1.5 mL of hydrogen peroxide were added. Microwave digestion was achieved using the microwave oven technique. The digested samples were quantitatively transferred into disposable flasks, and the necessary dilutions were made. Quantitation was performed using a five‐point calibration curve obtained using external element standards.

### 2.7 Determination of pesticide levels in propolis samples by LC–MS/MS

One ml of acetonitrile was added to 0.5 g of the sample and mixed using a vortex (Reax top, Heidolph, Germany). The mixture was sonicated at 45°C for 15 min and centrifuged at 9000 rpm. The supernatant was added to an Eppendorf tube containing 150 mg of MgSO_4_ and 50 mg of PSA and vortexed. The mixture was centrifuged at 13,500 rpm (Allegra X‐30, Beckman Coulter, USA) for 5 min, and the clear supernatant was filtered through a 0.22 μm nylon filter, added to a glass vial, and injected into the LC–MS/MS system for the analysis. An Agilent Poroshell 120 EC‐C18 LC column (3.0 × 50 mm, 2.7 μm) was used. Mobile phase A was 5 mM ammonium formate + 0.1% aqueous formic acid solution, and Mobile phase B was 0.1% formic acid solution in acetonitrile. The gradient program was as follows: Mobile phase A: 95% and Mobile phase B: 5% for 6 min; Mobile phase A: 5% and Mobile phase B: 95% for 3 min; Mobile phase B: 100% for 1.9 min; Mobile phase A: 95% and Mobile phase B: 5% for 0.1 min. Two MRM transitions were used. The method parameters for the pesticides are shown in Table S2. Gas flow was 10 L min^−1^ with a temperature of 325°C. Methanol was used for the preparation of the stock‐standard solutions of the pesticides. The stock solution of pesticides (10 mgL^−1^) was used to prepare the standard solutions to generate the calibration curves using six data points (1, 5, 10, 25, 50, and 100 μg L^−1^).

### 2.8 Calculation of the removal/reduction/loss of elements, pesticide residues, and phenolics after wax separation

The wax content of the W samples was assumed to be 100%, and that of the PF samples was assumed to be 0%, and these assumptions were taken into account in the further calculations. The percentage of removal of toxic elements (%) or loss of others (%) in CP samples through W samples after wax separation was calculated by using the ratio of the element content (as mg kg^−1^) in the W samples to the element content (as mg kg^−1^) in the corresponding CP samples, taking into account the percentage of the corresponding propolis fraction percentage in the raw sample, multiplied by 100. The percentage of elements remaining in the PF samples after wax separation was also calculated by using the ratio of the element content (as mg kg^−1^) in the PF samples to the element content (as mg kg^−1^) in the corresponding CP samples, taking into account the percentage of the corresponding propolis fraction percentage in the raw sample, multiplied by 100, and then these values were subtracted from 100% in order to calculate the reduction of the toxic elements (%) or the loss of the others (%) in PF samples. The reduction/removal of pesticide residues and the loss of total phenolics after wax separation were also calculated using both approaches. The mean reduction/removal/loss (%) in CP samples value was the mean value for three sample groups and was given with the standard deviation (STD) along with the relative standard deviation (RSD) values. Regarding the pesticide residue results, the removal/reduction percentages (%) were calculated only for the pesticides that were detected above the LOQs in at least two sample groups.

### 2.9 Statistical analysis

All measurements were done in triplicate, and the statistical analysis of the data was conducted using the PASW Statistics 18 software (SPSS Inc., IL, USA). Significant differences were determined by a one‐way analysis of variance (ANOVA). Means were compared using Duncan's multiple range test with a significance level of *p* < 0.05.

## 3 RESULTS AND DISCUSSION

### 3.1 The wax content of samples

The samples from different beekeepers were used in this study to consider a possible range of variability in the wax content of the crude propolis samples. The wax contents of the samples are shown in Table 1. The wax contents of crude samples were determined to be 81%, 55%, and 42%. The wax content was reported to be approximately 30%–40% for propolis by Silici (2008). Aksoy et al. (2017) also reported the average wax content in raw Turkish propolis, with 41% having the minimum and maximum values of 5.3 and 75.9% (Aksoy et al., 2017). Hence, the wax percentages of the samples were in accordance with the previous results. CP1 and CP2 samples had higher wax percentages than the average wax values in propolis, suggesting that the samples contain higher amounts of extrinsic wax. It is known that if raw propolis contains high amounts of extrinsic wax, it is washed with water (Burdock, 1998). The high wax percentage was even visually understandable in the CP1 sample. However, even this sample was not subjected to the wax separation process and was used directly in the study. So, the wide range of wax percentages in the samples desired for this study was achieved.

**TABLE 1:** The wax and the total phenolics content (TPC) of samples.

	Samples^1^								
	W1	PF1	CP1	W2	PF2	CP2	W3	PF3	CP3
Wax content (%)	100^2^	0^2^	81	100^2^	0^2^	55	100^2^	0^2^	42
TPC^3^ (mg g ^−1^ sample)	10.20^c^ ± 0.30	162.09^a^ ± 1.24	45.00^b^ ± 2.66	6.06^c^ ± 0.44	70.89^a^ ± 0.97	40.94^b^ ± 2.61	4.65^c^ ± 1.26	54.97^a^ ± 0.78	31.90^b^ ± 2.06

^1^ CP, W, and PF indicate crude propolis, wax fraction, and remaining propolis fraction after the wax separation, respectively.

^2^ The wax content of the W samples was assumed as 100%, and that of PF samples was assumed as 0%.

^3^ The results are given as mean ± STD (*n* = 3). The mean TPC values of the corresponding sample group with different superscript letters (a, b and c) are significantly different (*p* < .05).

It is known that the wax percentage of crude propolis is important since it affects the balsam percentage. A low percentage of wax and insoluble matter means that the propolis sample contains a high percentage of balsam, which is an influential portion of the biological activity (Popova et al., 2007). However, as can be seen from the results of the present study (Table 1), not only the amount of wax percentage but also the amount of active compounds (such as phenolics) in the remaining portion after wax separation is of great importance. Despite having the highest wax content, the phenolic content was also the highest in the CP1 sample.

### 3.2 Phenolic compounds of samples

The TP content of the samples was calculated using the calibration curve (*R* ^*2*^ = .995) obtained by taking the dilution factors into account. As can be seen from the TP content of the samples in Table 1, the total phenolic content of the CP samples was determined to be in the range of 31.90–45.00 mg GAE g ^−1^ and that of the PF samples was in the range of 54.97–162.09 mg GAE g^−1^. These values are in accordance with the previous findings. Ozdal et al. (2019) reported the total phenolic content of 11 raw propolis samples collected from various geographical areas in Turkey in the range of 27.49–199.70 mg GAE g^−1^.

The effect of the separation of the wax from the crude propolis can be clearly observed from the TP content results. The wax portions (W samples), having TP contents varying between 4.65 and 10.20 mg GAE g^−1^, do not contain a substantial amount of phenolics. It can be seen from the results that the W samples had significantly (*p* < .05) lower TP content than the corresponding sample groups (i.e. the PF and the CP samples). As can be expected, separation of the wax portion from the crude propolis increased the total phenolics in the propolis fraction (PF samples).

The main biologically active components of propolis were reported to be the phenolic compounds, including the flavonoids, together with some others such as esters and terpenes (Rufatto et al., 2018). Hence, some phenolic compounds of propolis fractions were also investigated by LC–MS/MS analysis, as seen in Table 2. The recovery rates for 13 compounds studied (*p*‐coumaric acid, 3,4 dihydroxybenzoic acid, quercetin, caffeic acid, *t*‐ferulic acid, chlorogenic, gallic acid, epicathechin, pyracathechol, sinapic acid, vanilic acid, syringic acid, phlorizin) were detected in the range of 102%–84% for propolis, 103%–70% for wax, and 93%–76% for crude propolis samples, respectively. The compounds detected in the samples are given in Table 2. PF1 and CP1 samples contain higher amounts of phenolics detected (Table 1), and hence the results were in accordance with the TP results (Table 2). The abundant phenolics detected in the samples were caffeic acid, *t*‐ferulic acid, and *p*‐coumaric acids in the ranges of 0.665–5.054, 0.886–5.526, and 0.218–1.148 mg g^−1^ in CP samples, respectively. Ozdal et al. (2018) reported the mean values for the concentrations of caffeic acid, ferulic acid, *t*‐cinnamic acid, and *p*‐coumaric acid as 0.88, 0.56, 0.51, and 0.49 mg kg^−1^ in Turkish propolis, besides the high concentrations of flavonoids such as chrysin, pinobanksin, pinostrobin, galangin, and pinocembrin. Chrysin, galangin, pinocembrin, and pinobanksin (and its esters) as the most abundant flavonoids and caffeic acid derivatives, followed by *p*‐coumaric acid derivatives and ferulic and isoferulic acid as the abundant phenolic acids, were reported as the typical constituents for propolis from temperate zones, with *Populus* spp. as a plant source (Pellati et al., 2011). Chrysin, galangin, pinocembrin, and pinobanksin were not studied in the present study. Among the flavonoids studied in the present study, only quercetin was detected in the concentration ranges of 0.213–0.653 mg g^−1^. It is known that the chemical composition, especially the total phenolic contents and individual phenolic profiles of propolis samples, depends on the geographical origin (Ozdal et al., 2019) and, accordingly, the plant origin (Çelemli & Sorkun, 2012). Even among the samples obtained from the same city, differences in the botanical origin of the propolis samples were reported (Çelemli & Sorkun, 2012).

**TABLE 2:** Phenolic compounds detected in samples.

Compound	Phenolic compounds detected in samples^1^ ^,^ ^2^ (mg g^−1^)								
	W1	PF1	CP1	W2	PF2	CP2	W3	PF3	CP3
Quercetin	0.047^c^ ± 0.002	0.681^a^ ± 0.011	0.653^b^ ± 0.007	0.031^c^ ± 0.004	0.525^a^ ± 0.010	0.213^b^ ± 0.003	0.120^c^ ± 0.009	0.445^a^ ± 0.005	0.383^b^ ± 0.005
Epicathechin	–	–	–	–	–	–	–	–	–
Pyracathechol	–	–	–	–	–	–	–	–	–
Phlorizin	–	–	–	–	–	–	–	–	–
*p*‐Coumaric acid	0.209^c^ ± 0.003	1.753^a^ ± 0.193	1.148^b^ ± 0.012	0.176^c^ ± 0.004	1.084^a^ ± 0.002	0.459^b^ ± 0.010	0.086^c^ ± 0.003	0.606^a^ ± 0.002	0.218^b^ ± 0.002^b^
3,4 Dihydroxybenzoic acid	0.007^c^ ± 0.001	0.180^a^ ± 0.001	0.048^b^ ± 0.002	0.010^c^ ± 0.000	0.136^a^ ± 0.002	0.036^b^ ± 0.001	0.025^c^ ± 0.001	0.131^a^ ± 0.001	0.049^b^ ± 0.002
Caffeic acid	0.480^c^ ± 0.013	8.874^a^ ± 0.094	5.054^b^ ± 0.046	0.296^c^ ± 0.0180	1.943^a^ ± 0.002	1.536^b^ ± 0.006	0.282^c^ ± 0.007	1.216^a^ ± 0.001	0.665^b^ ± 0.005
*t*‐Ferrulic acid	0.421^c^ ± 0.003	7.668^a^ ± 0.070	5.526^b^ ± 0.026	0.307^c^ ± 0.002	3.322^a^ ± 0.002	1.100^b^ ± 0.098	0.382^c^ ± 0.007	3.919^a^ ± 0.007	0.886^b^ ± 0.014
Chlorogenic acid	–	–	–	–	–	–	–	–	–
Gallic acid	–	–	–	–	–	–	–	–	–
Sinapic acid	–	–	–	–	–	–	–	–	–
Vanilic acid	–	–	–	–	–	–	–	–	–
Syringic acid	–	–	–	–	–	–	–	–	–

*Note*: –, Not determined (<LOD).

^1^ The mean concentrations in each sample group with different superscript letters (a, b and c) are significantly different (*p* < .05).

^2^ CP, W, and PF indicate crude propolis, wax fraction, and remaining propolis fraction after the wax separation, respectively.

### 3.3 Elemental composition and toxic elements in the samples

The elements detected in the propolis samples are given in Table 3. CP samples contain K (347.386–524.337 mg kg^−1^), Ca (269.573–396.082 mg kg^−1^), Si (130.079–178.669 mg kg^−1^), Mg (34.813–193.594 mg kg^−1^), Fe (61.892–137.629 mg kg^−1^), Al (34.108–132.322 mg kg^−1^), and Zn (19.375–40.281 mg kg^−1^) in relatively higher amounts than the other elements. The concentrations for the elements other than Na (31.642–40.670 mg kg^−1^), B (2.476–7.916 mg kg^−1^), Mn (2.746–4.754 mg kg^−1^), and Ni (7.314–11.536 mg kg^−1^) were in values near 1 mg kg^−1^ or lower. The values reported in the present study were generally in agreement with the data of the previous studies reporting the elemental composition of propolis (Dogan et al., 2006; Golubkina et al., 2016). Aksoy et al. (2017) also reported similar elements in Turkish propolis. Since propolis is collected from various plants in various areas, the wide range of elemental composition of the propolis samples could be explained by the different mineral composition of the plants, which is influenced by numerous environmental factors (Aksoy et al., 2017; Cvek et al., 2008).

**TABLE 3:** Concentrations of elements in samples.

	Samples^1^ ^,^ ^2^										
	W1	PF1	CP1	W2	PF2	CP2	W3	PF3	CP3	PF4^3^	CP4^3^
Elements (mg kg^−1^)											
Be	0.005 ± 0	0.009 ± 0	0.005 ± 0	0.004 ± 0.001	0.01 ± 0.003	0.005 ± 0	0.001 ± 0	0.005 ± 0	0.005 ± 0	0.005 ± 0.001	0.003 ± 0
B	8.488^a^ ± 0.01	5.642^b^ ± 0.045	7.916^c^ ± 0.042	5.571^c^ ± 0.17	15.223^a^ ± 0.163	6.93^b^ ± 0.057	0.349^c^ ± 0.008	2.899^a^ ± 0.077	2.476^b^ ± 0.003	1.031 ± 0.082	0.149 ± 0.002
Na	21.339^c^ ± 0.158	85.165^a^ ± 1.658	40.670^b^ ± 0.146	26.619^c^ ± 0.082	64.738^a^ ± 0.083	35.377^b^ ± 0.538	17.119^c^ ± 0.163	45.273^a^ ± 0.007	31.642^b^ ± 0.047	26.546 ± 0.327	31.104 ± 0.082
Mg	18.529^c^ ± 0.303	173.593^a^ ± 0.319	67.531^b^ ± 1.653	21.672^c^ ± 0.245	377.243^a^ ± 0.152	193.594^b^ ± 2.935	22.065^c^ ± 0.082	56.233^a^ ± 0.741	34.813^b^ ± 1.961	59.096 ± 0.445	38.278 ± 0.082
Al	18.179^c^ ± 0.157	191.477^a^ ± 0.121	53.916^b^ ± 0.248	18.326^c^ ± 0.082	267.419^a^ ± 30.297	132.322^b^ ± 5.801	13.493^c^ ± 0.087	55.631^a^ ± 0.129	34.108^b^ ± 0.64	46.541 ± 0.108	36.295 ± 0.017
Si	78.918^c^ ± 0.018	214.583^a^ ± 0.541	130.079^b^ ± 0.483	68.371^c^ ± 0.49	310.688^a^ ± 8.429	174.61^b^ ± 0.01	102.145^c^ ± 0.163	147.699^b^ ± 1.762	178.669^a^ ± 0.082	125.111 ± 0.031	93.551 ± 0.327
K	159.827^c^ ± 3.121	995.636^a^ ± 4.447	454.714^b^ ± 0.601	152.421^c^ ± 0.169	1290^a^ ± 0	524.337^b^ ± 17.607	234.984^c^ ± 0.016	367.996^a^ ± 0.297	347.386^b^ ± 0.175	416.689 ± 0.49	325.388 ± 0.49
Ca	348.655^a^ ± 42.526	352.992^a^ ± 3.276	396.082^a^ ± 0.333	261.956^c^ ± 0.082	394.847^a^ ± 0.923	311.486^b^ ± 7.196	242.808^c^ ± 0.224	357.617^a^ ± 0.653	269.573^b^ ± 0.082	298.263 ± 0.082	299.124 ± 0.572
V	0.811^a^ ± 0.036	0.713^b^ ± 0.032	0.693^b^ ± 0.018	0.249^c^ ± 0.015	1.15^a^ ± 0.086	0.604^b^ ± 0.061	0.251^c^ ± 0.036	0.541^a^ ± 0.019	0.341^b^ ± 0.007	0.27 ± 0.024	0.25 ± 0.082
Cr	1.32^b^ ± 0.004	1.736^a^ ± 0.02	1.362^b^ ± 0.04	0.976^b^ ± 0.024	0.906^b^ ± 0.065	1.292^a^ ± 0.131	0.609^b^ ± 0.073	1.063^a^ ± 0.029	0.728^b^ ± 0.041	0.53 ± 0.057	1.462 ± 0.033
Mn	1.727^c^ ± 0.02	6.068^a^ ± 0.006	2.746^b^ ± 0.219	1.548^c^ ± 0.032	2.207^b^ ± 0.193	2.942^a^ ± 0.046	1.658^b^ ± 0.033	5.284^a^ ± 0.49	4.754^a^ ± 0.033	3.915 ± 0.163	3.336 ± 0.016
Fe	28.079^c^ ± 0.072	142.942^a^ ± 0.478	61.892^b^ ± 0.293	48.845^c^ ± 0.163	287.652^a^ ± 0.434	137.629^b^ ± 3.44	70.305^a^ ± 0.49	34.778^c^ ± 1.236	65.829^b^ ± 0.082	63.284 ± 0.068	59.429 ± 0.074
Co	0.14^b^ ± 0.002	0.193^a^ ± 0.009	0.125^b^ ± 0.026	0.049^c^ ± 0.012	0.153^a^ ± 0.006	0.105^b^ ± 0.009	0.061^a^ ± 0.007	0.066^a^ ± 0.001	0.053^a^ ± 0.008	0.055 ± 0.004	0.082 ± 0.002
Ni	13.325^a^ ± 0.351	9.132^c^ ± 0.45	11.536^b^ ± 0.073	3.892^b^ ± 0.141	7.032^a^ ± 0.162	7.314^a^ ± 0.236	0.475^c^ ± 0.02	14.346^a^ ± 0.25	9.25^b^ ± 0.041	4.587 ± 0.033	21.011 ± 0.088
Cu	0.803^b^ ± 0.033	0.111^c^ ± 0.005	0.936^a^ ± 0.036	1.732^a^ ± 0.053	1.313^b^ ± 0.009	1.637^a^ ± 0.053	0.293^c^ ± 0.016	0.847^b^ ± 0.005	0.941^a^ ± 0.02	0.573 ± 0.022	0.605 ± 0.001
Zn	25.495^a^ ± 0.978	22.083^b^ ± 0.554	19.375^c^ ± 0.183	27.688^c^ ± 0.07	38.955^b^ ± 0.056	40.281^a^ ± 0.572	21.539^b^ ± 0.028	18.141^c^ ± 0.615	28.367^a^ ± 0.033	16.536 ± 0.016	24.128 ± 0.108
As	0.341^a^ ± 0.016	0.288^b^ ± 0.026	0.377^a^ ± 0.001	0.205^c^ ± 0.008	0.489^a^ ± 0.004	0.307^b^ ± 0.068	0.227^b^ ± 0.007	0.267^a^ ± 0.017	0.258^a^ ± 0.008	0.21 ± 0.002	0.2 ± 0.015
Cd	0.015^ab^ ± 0.002	0.02^a^ ± 0.003	0.012^b^ ± 0.001	0.007^a^ ± 0.001	0.003^c^ ± 0	0.005^b^ ± 0	0.007^b^ ± 0	0.014^a^ ± 0	0.012^a^ ± 0.002	0.015 ± 0.001	0.015 ± 0.004
Tl	0.001 ± 0	0.002 ± 0	0.001 ± 0	0^c^ ± 0	0.002^a^ ± 0	0.001^b^ ± 0	0.001 ± 0	0.001 ± 0	0.001 ± 0	0.001 ± 0	0 ± 0.001
Pb	0.508^c^ ± 0.006	1.201^a^ ± 0.017	0.585^b^ ± 0.004	0.345^c^ ± 0.011	1.52^a^ ± 0.02	0.736^b^ ± 0.008	0.326^a^ ± 0.021	0.232^a^ ± 0	0.225^a^ ± 0.072	0.293 ± 0	0.357 ± 0.006

^1^ The results are given as mean ± STD (*n* = 3). The mean element concentrations in each sample group with different superscript letters (a, b and c) are significantly different (*p* < .05).

^2^ CP, W, and PF indicate crude propolis, wax fraction, and remaining propolis fraction after the wax separation, respectively.

^3^ PF4 and CP4 samples were obtained from a non‐agricultural place to be used in recovery studies.

It is known that environmental pollution could be effective in the presence of toxic elements such as, Cd, Hg, and Pb in propolis (Cvek et al., 2008; Golubkina et al., 2016). In the present study, As, Cd, and Pb were detected in some samples, as shown in Table 3. The Cd values of the CP samples were between 0.005 and 0.012 mg kg^−1^. As values of the CP samples were determined to be in the range of 0.258–0.377 mg kg^−1^. In the Croatian propolis samples analyzed, Hg and As levels were determined between 0.003–0.053, and 0.039–3.020 mg kg^−1^, respectively (Cvek et al., 2008). Similar As and Cd values were reported for propolis samples from Moldavia (Golubkina et al., 2016). Turkish propolis samples were also reported to contain toxic metals in limited concentrations. Maximum levels of As and Cd detected in the Turkish propolis samples were reported as 0.573 and 2.441 mg kg^−1^, respectively (Aksoy et al., 2017). The values of these toxic metals obtained in the present study were lower than the reported values (Aksoy et al., 2017; Cvek et al., 2008).

As can be seen in Table 3, Pb, a toxic metal that has serious effects on human health, is the most prominent of the toxic elements analyzed. Crude propolis samples, namely CP1, CP2, and CP3, contained Pb at values of 0,585, 0,736, and 0,225 mg kg^−1^, respectively. Pb values of PF1, PF2, and PF3 were in the range of 0.232–1.520, and mg kg^−1^. These results are consistent with the findings of Aksoy et al. (2017), who reported Pb values ranging from 0.023 to 0.843 mg kg^−1^ in Turkish propolis samples. These Pb values obtained for Turkish propolis samples are relatively low compared to the published data. Gonzalez‐Martin et al. (2018) revealed the presence of toxic metals such as Cr, Ni, Cu, Zn, and Pb (Gonzalez‐Martin et al., 2018). They highlighted the occurrence of Pb at levels higher than 0.1 mg kg^−1^ in the commercially processed propolis samples analyzed. In view of the health and safety of propolis, Bogdanov (2006) and Cvek et al. (2008) also reported Pb as the main contamination risk among toxic elements (Bogdanov, 2006; Cvek et al., 2008). Golubkina et al. (2016) reported Pb levels in propolis samples from Moldavia in the range of 1.52–16.07 mg kg^−1^. Sales et al. (2006) reported Pb levels of 2 mg kg^−1^ and 8 mg kg^−1^ for different propolis harvesting methods. Cvek et al. (2008) found the Pb content of the propolis between 0.314 and 64.020 mg kg^−1^. Among the raw propolis samples they analyzed, two of them did not meet the WHO standards for the maximum permitted Pb content (10 mg kg^−1^). The heavily contaminated sample (64.020 mg kg^−1^) was reported as the sample originating from one of the most polluted industrial centers in Croatia (Cvek et al., 2008), as can be expected. In fact, the main reason for the presence of toxic metals such as Pb in bee products is that the origin of the samples is in areas with high environmental pollution due to their proximity to highways or industrial areas.

### 3.4 The effect of wax separation on pesticide residues in propolis

Propolis has a complex structure depending on its geographic origin. Pesticides can be found in materials produced in hives, such as honey, propolis, and beeswax. In this study, the pesticide levels of wax, propolis, and crude propolis samples were determined using the LC–MS/MS method. The concentrations of pesticide levels in samples are shown in Table 4. Different types of pesticides, including fungicides (carbendazim, thiophanate‐methyl, metalaxyl, fenpropimorph, spiroxamine, azoxystrobin, tebuconazole, and trifloxystrobin), herbicides (tebuthiuron, secbumeton), insecticides (thiamethoxam, acetamiprid), and acarides (hexythiazox, propargite) were studied. Among the pesticides studied, seven of them, i.e. carbendazim, metalaxyl, tebuconazole, thiamethoxam, acetamiprid, hexythiazox, and propargite, were detected in at least one sample above the level of 1 μg kg^−1^. The maximum residue limit (MRL) value of carbendazim (carbendazim and thiophanate‐methyl, expressed as carbendazim) applied to “Honey and other apiculture products” is 1000 μg kg^−1^ according to EU legislation. The MRL value of propargite, thiamethoxam, metalaxyl, tebuconazole applied to the aforementioned group is 50 μg kg^−1^. Hexythiazox has an MRL value of 20 μg kg^−1^ for these products. Hence, the detected residue levels for all samples were below the corresponding MRLs (https://ec.europa.eu/food/plant/pesticides/eu‐pesticides‐database).

**TABLE 4:** Concentrations of pesticides in samples.

Pesticides detected in samples^1^ (μg kg^−1^)											
Pesticide residue	W1	PF1	CP1	W2	PF2	CP2	W3	PF3	CP3	PF4^3^	CP4^3^
Carbendazim	3.71	9.96	9.69	11.92	12.53	12.33	1.64	1.29	2.29	<LOQ	<LOQ
Thiamethoxam	<LOQ^2^	<LOQ	<LOQ	<LOQ	1.19	1.34	<LOQ	<LOQ	<LOQ	–	–
Acetamiprid	<LOQ	<LOQ	<LOQ	1.04	<LOQ	<LOQ	–	–	–	–	–
Tebuthiuron	–	–	–	–	–	–	–	–	–	–	–
Secbumeton	–	–	–	–	–	–	–	–	–	–	–
Thiophanate Methyl	–	–	–	–	–	–	–	–	–	<LOQ	<LOQ
Metalaxyl	3.00	1.64	2.16	3.55	2.59	2.78	1.74	<LOQ	1.18	–	–
Fenproprimorph	<LOQ	<LOQ	<LOQ	<LOQ	<LOQ	<LOQ	<LOQ	<LOQ	<LOQ	–	–
Spiroxamine	<LOQ	<LOQ	<LOQ	<LOQ	<LOQ	<LOQ	–	–	–	–	–
Azoxystrobin	–	–	–	–	–	–	–	–	–	–	–
Tebuconazole	3.14	1.38	4.13	2.46	1.58	1.93	–	–	–	–	–
Trifloxystrobin	<LOQ	<LOQ	<LOQ	<LOQ	<LOQ	<LOQ	–	–	–	–	–
Hexythiazox	2.05	<LOQ	<LOQ	–	–	–	–	–	–	–	–
Propargite	23.70	1.40	19.65	2.55	1.28	1.77	–	–	–	–	–

*Note*: –, Not determined (<LOD).

^1^ PW, W, and P indicate crude propolis, wax fraction, and remaining propolis fraction after the wax separation, respectively.

^2^ LOQ was determined as 1 μg kg^−1^.

^3^ PF4 and CP4 samples were obtained from a non‐agricultural place to be used in recovery studies.

Carbendazim was the only pesticide detected in all samples among the pesticides studied, albeit at low levels. Carbendazim is known as a fungicide that was widely used in order to prevent the fungal infections of agricultural products. However, its use is not allowed under the current legislation in Turkey and the EU. Metalaxyl is a systemic benzoid fungicide (Pose‐Juan et al., 2009). Tebuconazole is also a fungicide, which was detected in the range of 1.93–4.13 μg kg^−1^ in the CP samples. There were some other pesticides detected in the samples, including thiamethoxam, acetamiprid, and hexythiazox, which belong to insecticides. These pesticides are known to be used for the control of insects that damage the products. The concentration of propargite was detected at 23.70 μg kg ^−1^ in sample W1, which was the highest amount among the concentrations of all detected pesticides. Propargite is an acaricide (Luo et al., 2014). In the literature, some studies are reporting propargite residues in bee products. In a study, Lozano et al. (2019) evaluated residues in honey, beeswax, and bee bread. The propargite residue was reported to be detected as 19 μg kg ^−1^ in beeswax (Lozano et al., 2019). There are also some studies concerning the pesticide residues in propolis. In the study of Gonzalez‐Martin et al. (2018), the presence of pesticides, including fungicides such as triadimefon, procymidone, dichlofluanid, and folpet, herbicides such as metazachlor, and acaricides such as chlorfenson, in propolis was reported. The fungicide triadimefon was reported to be detected in 56.3% of the processed products analyzed (Gonzalez‐Martin et al., 2018). Another study investigated the determination of organophosphate pesticides like ethion, coumaphos, and chlorpyrifos, which can be found in trace amounts in ethanolic propolis extracts (Pérez‐Parada et al., 2011). Calatayud‐Vernich et al. (2018) also investigated the pesticide residues in honeybees, beeswax, and pollen collected from different regions of Spain. They found that the most common pesticide group in beekeeping matrices was acaricides such as coumaphos, fluvalinate, hexythiazox, and 2,4‐dimethylphenylformamide. They also emphasized that most of the pesticides detected were the ones used in beekeeping and agricultural practices (Calatayud‐Vernich et al., 2018). Morales et al. (2020) detected pesticide residues in beeswax, beebread, and bee brood. They detected 31 different pesticides, including fungicides (fluopyram, iprovalicarb, epoxiconazole), acaricides (pyridaben, dimethoate), insecticides (imidacloprid, chlorantraniliprole, chlorpyrifosmethyl), and herbicides (pendimethalin, oxasulfuron) (Morales et al., 2020).

The results of the present study showed that the propolis fractions might contain pesticide residues in various concentrations. To the best of our knowledge, there is no study on the transition of pesticides from raw propolis to extracts. It can be predicted that the extraction procedures might be influential in the concentration of pesticides in the propolis extracts, like in the example of elements (Cvek et al., 2008), and this might also reduce the concentration of pesticides in the corresponding extract. Even so, the amount of pesticide residue in raw propolis is important.

### 3.5 The effect of wax separation on the removal of toxic elements, pesticides, and the loss of essential elements and phenolics

**FIGURE 1:**
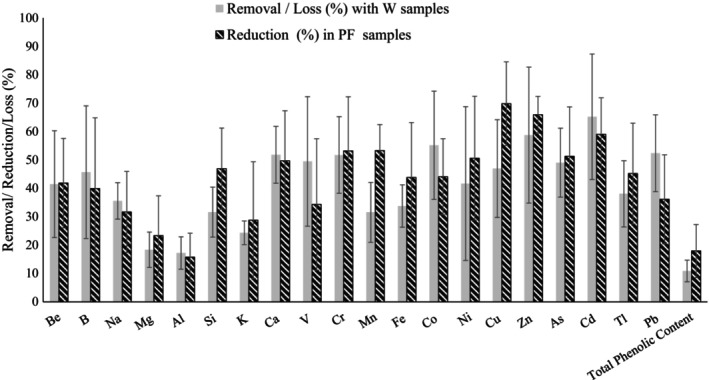
Removal/reduction of elements and loss/reduction of total phenolics through wax separation. The results are given as mean ± standard error of mean regarding the three different sample group (*n* = 3). W and PF indicate wax fraction and propolis fraction after the wax separation, respectively.

In order to understand the effect of wax separation on the removal of toxic elements and the loss of essential elements and phenolics, the removal/loss percentages were calculated and given in Table 5. The mean values obtained using three different crude propolis samples were given along with the standard deviation (STD) and the relative standard deviation (RSD) values. The removal/reduction/loss (%) percentages obtained using both approaches can also be seen in Figure 1. Al removal was calculated at 17.18% on the basis of W samples and 15.66% on the basis of PF samples. These values indicate that wax separation was not good at removing Al. However, other toxic metals such as Cd and Pb were successfully removed from the samples (65.18% or 59.00% of Cd and 52.34% or 36.10% of Pb were removed). Almost 51.73% or 53.16% of Cr and 49.00% or 51.27% of As were also removed by this method. The variations in the removal values obtained for different elements may be because of the differences in the composition of the propolis samples or the alterations in the solubility of the elements with pH (Roberson & Hem, 1969). Unfortunately, due to the lack of published data, we were unable to compare our results with the results of other studies.

**TABLE 5:** Removal/loss of elements, pesticides, and total phenolics through wax separation.

	Removal/Reduction/loss (%)					
	Calculation on the basis of W samples			Calculation on the basis of PF samples		
	Mean	STD	RSD	Mean	STD	RSD
Elements						
Be	41.44	32.60	0.79	41.81	27.31	0.65
B	45.66	40.48	0.89	39.89	43.19	1.08
Na	35.54	11.11	0.31	31.63	24.76	0.78
Mg	18.33	10.77	0.59	23.26	24.35	1.05
Al	17.18	9.86	0.57	15.66	14.72	0.94
Si	31.56	15.27	0.48	46.88	24.77	0.53
K	24.29	7.19	0.30	28.75	35.58	1.24
Ca	51.80	17.41	0.34	49.69	30.57	0.62
V	49.46	39.52	0.80	34.30	40.08	1.17
Cr	51.73	23.41	0.45	53.16	33.04	0.62
Mn	31.51	18.29	0.58	53.27	15.90	0.30
Fe	33.71	12.94	0.38	43.81	33.45	0.76
Co	55.13	33.06	0.60	44.05	23.21	0.53
Ni	41.66	46.95	1.13	50.58	37.84	0.75
Cu	46.93	29.84	0.64	69.80	25.52	0.37
Zn	58.76	41.52	0.71	65.91	11.24	0.17
As	49.00	21.07	0.43	51.27	30.18	0.59
Cd	65.18	38.32	0.59	59.00	22.38	0.38
Tl	38.06	20.20	0.53	45.17	30.77	0.68
Pb	52.34	23.48	0.45	36.10	27.17	0.75
Pesticides						
Carbendazim	38.09	13.10	0.34	67.35	13.11	0.19
Metalaxyl	81.57	26.99	0.33	72.67	13.84	0.19
Tebuconazole	65.99	6.16	0.09	78.36	21.65	0.28
Propargite	88.46	13.04	0.15	83.05	22.05	0.27
Total phenolic content	10.88	6.55	0.60	17.89	16.17	0.90

Abbreviations: RSD, relative standard deviation; STD, standard deviation (*n* = 3).

The percentages of removal of pesticides were also calculated and given in Table 5. In this study, although it was a positive result that the pesticides detected had low concentrations or even below the LODs and LOQs, this did not allow us to study the distribution of all pesticides detected among fractions (PF, W, and CP samples). In order to evaluate the effect of the wax separation on the pesticide residue results, the percentages of removal/reduction (%) of pesticides were only calculated for the ones that were detected above the LOQs in at least two sample groups. As can be seen from Table 4, these were carbendazim, metalaxyl, tebuconazole, and propargite. Removal of significant amounts of carbendazim (38.09%–67.35%), metalaxyl (81.57%–72.67%), tebuconazole (65.99%–78.36%), and propargite (88.46%–83.05%) was achieved by wax separation.

**FIGURE 2:**
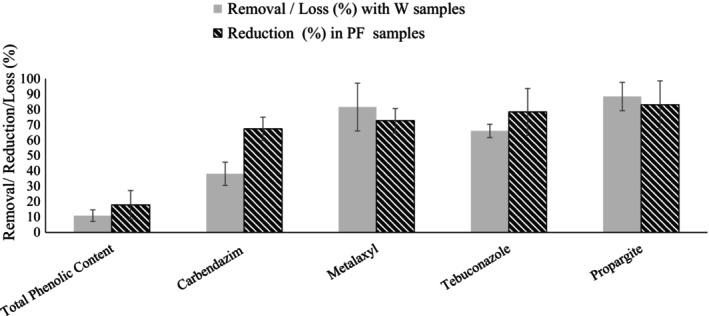
Removal/reduction of pesticide residues and loss/reduction of total phenolics through wax separation. The results are given as mean ± standard error of mean regarding the three different sample group (*n* = 3). W and PF indicate wax fraction and propolis fraction after the wax separation, respectively.

The percentage of phenolics lost (L%) in CP samples through W samples and the percentage reduction of phenolics in the PF samples after wax separation are also given in Table 5. Loss/reduction values of 10.88% and 17.89% were calculated for phenolics. Besides the high percentages of removal/reduction values obtained for pesticide residues and toxic elements, the loss/reduction percentages of total phenolics through wax separation were relatively low (Figures 1 and 2). In order to remove some toxic contaminants, a certain level of phenolic compound loss should be considered acceptable. The process of wax separation allows the removal of toxic substances from propolis without causing huge losses in phenolics.

In conclusion, one should bear in mind that only a part of the total amount of the contaminant in the raw propolis is transferred to the extracts, like in the example of toxic elements (Cvek et al., 2008), and this will reduce the amount of contaminants in the corresponding extract. Nevertheless, the concentration of contaminants in raw propolis that will be subjected to extraction prior to usage, is of great importance. Hence, the removal of the wax portion could serve as a valuable process step for the reduction/ removal of contaminants.

## 4 CONCLUSIONS

Propolis is a natural product containing not only valuable components but also some possible contaminants. The effect of wax separation on macro‐ and trace elements, toxic elements, and pesticide residues, besides the phenolic compounds of propolis, was studied.

Even though the samples in the current study had low levels of toxic elements, care should be given to the toxic elements in the propolis samples because some of the toxic elements could also be present in the propolis fractions, including Pb, As, and Cd. The process of wax separation allows the removal of some toxic elements and pesticides from propolis without causing huge losses in phenolics. Removal of significant amounts of Cr, Pb, As, and Cd and pesticides including carbendazim, metalaxyl, tebuconazole, and propargite was achieved by wax separation. Hence, the removal of the wax portion could serve as a valuable process step for the reduction/removal of the toxic contaminants in crude propolis.

The results of the present study provide a brief assessment of the effects of wax separation on the components of propolis, with a perspective of detecting valuable constituents as well as some contaminants. Based on the results of the present study, further research should be conducted in order to characterize the distribution of potential contaminants in the propolis fractions and also in the extracts of propolis. The levels of contaminants in the raw propolis that will be subjected to extraction prior to usage are of great importance. In order to ensure the safety of propolis products, which are faced with an increasing demand by consumers worldwide, especially due to the search for health‐promoting products, studies on possible toxic contaminants in both raw and processed propolis products should be increased.

## AUTHOR CONTRIBUTIONS

**Eylul Evran:** Conceptualization (equal); data curation (equal); formal analysis (equal); methodology (equal); resources (equal); writing – original draft (equal). **Serap Durakli‐Velioglu:** Conceptualization (equal); data curation (equal); formal analysis (equal); methodology (equal); project administration (equal); resources (equal); validation (equal); writing – original draft (equal); writing – review and editing (equal). **Hasan Murat Velioglu:** Conceptualization (equal); data curation (equal); formal analysis (equal); methodology (equal); resources (equal); writing – original draft (equal); writing – review and editing (equal). **Ismail Hakki Boyaci:** Conceptualization (equal); project administration (equal); supervision (equal); validation (equal); writing – review and editing (equal).

## FUNDING INFORMATION

The authors received no financial support for the research, authorship, and publication of this article.

## CONFLICT OF INTEREST STATEMENT

The authors declare that they have no competing interests.

## Supporting information


Table S1

## Data Availability

The data that support the findings of this study are available from the corresponding author upon reasonable request.

## References

[fsn33866-bib-0001] Aksoy, C. , Atabay, M. M. , Tirasoglu, E. , Koparan, E. T. , & Kekillioglu, A. (2017). Elemental content profiles in propolis from several cities of Turkey. Functional Foods in Health and Disease, 7, 661–670.

[fsn33866-bib-0002] Amoros, M. , Simõs, C. M. O. , Girre, L. , Sauvager, F. , & Cormier, M. (1992). Synergistic effect of flavones and flavonols against herpes simplex virus type 1 in cell culture. Comparison with the antiviral activity of propolis. Journal of Natural Products, 55, 1732–1740. 1338212 10.1021/np50090a003

[fsn33866-bib-0003] Apaydin, H. , & Gümüş, T. (2018). Inhibitory effect of propolis (bee gum) against *Staphylococcus aureus* bacteria isolated from instant soups 1. Journal of Tekirdag Agricultural Faculty, 15, 67–75.

[fsn33866-bib-0004] Bankova, V. , Bertelli, D. , Borba, R. , Conti, B. J. , da Silva Cunha, I. B. , Danert, C. , Eberlin, M. N. , I Falcão, S. , Isla, M. I. , Moreno, M. I. N. , Papotti, G. , Popova, M. , Santiago, K. B. , Salas, A. , Sawaya, A. C. H. F. , Schwab, N. V. , Sforcin, J. M. , Simone‐Finstrom, M. , Spivak, M. , … Zampini, C. (2019). Standard methods for Apis mellifera propolis research. Journal of Apicultural Research, 58, 1–49.

[fsn33866-bib-0005] Bankova, V. S. , de Castro, S. L. , & Marcucci, M. C. (2000). Propolis: Recent advances in chemistry and plant origin. Apidologie, 31, 3–15.

[fsn33866-bib-0006] Banskota, A. H. , Tezuka, Y. , & Kadota, S. (2001). Recent progress in pharmacological research of propolis. Phytotherapy Research, 15, 561–571. 11746834 10.1002/ptr.1029

[fsn33866-bib-0007] Bayram, N. E. , Gerçek, Y. C. , Bayram, S. , & Toğar, B. (2020). Effects of processing methods and extraction solvents on the chemical content and bioactive properties of propolis. Journal of Food Measurement and Characterization, 14, 905–916.

[fsn33866-bib-0008] Blažková, I. , Hrouzek, J. , Szarka, A. , Pócsová, T. , & Hrouzková, S. (2022). Analytical methods for pesticide residues determination in propolis and propolis‐based products. Acta Chimica Slovaca, 15(1), 103–116.

[fsn33866-bib-0009] Bogdanov, S. (2006). Contaminants of bee products. Apidologie, 37, 1–18.

[fsn33866-bib-0010] Bueno‐Silva, B. , Alencar, S. M. , Koo, H. , Ikegaki, M. , Silva, G. V. J. , Napimoga, M. H. , & Rosalen, P. L. (2013). Anti‐inflammatory and antimicrobial evaluation of neovestitol and vestitol isolated from Brazilian red propolis. Journal of Agricultural and Food Chemistry, 61, 4546–4550. 23607483 10.1021/jf305468f

[fsn33866-bib-0011] Burdock, G. A. (1998). Review of the biological properties and toxicity of bee propolis (propolis). Food and Chemical Toxicology, 36, 347–363. 9651052 10.1016/s0278-6915(97)00145-2

[fsn33866-bib-0012] Calatayud‐Vernich, P. , Calatayud, F. , Simó, E. , & Picó, Y. (2018). Pesticide residues in honey bees, pollen and beeswax: Assessing beehive exposure. Environmental Pollution, 241, 106–114. 29803024 10.1016/j.envpol.2018.05.062

[fsn33866-bib-0013] Catchpole, O. J. , Grey, J. B. , Mitchell, K. A. , & Lan, J. S. (2004). Supercritical antisolvent fractionation of propolis tincture. Journal of Supercritical Fluids, 29, 97–106.

[fsn33866-bib-0014] Çelemli, Ö. G. , & Sorkun, K. (2012). The plant choices of honey bees to collect propolis in Tekirdag‐Turkey. Hacettepe Journal of Biology and Chemistry, 40, 45–51.

[fsn33866-bib-0015] Contieri, L. S. , de Souza Mesquita, L. M. , Sanches, V. L. , Chaves, J. , Pizani, R. S. , da Silva, L. C. , Viganó, J. , Ventura, S. P. M. , & Rostagno, M. A. (2022). Recent progress on the recovery of bioactive compounds obtained from propolis as a natural resource: Processes, and applications. Separation and Purification Technology, 298, 121640.

[fsn33866-bib-0016] Cvek, J. , Medić‐Šarić, M. , Vitali, D. , Vedrina‐Dragojević, I. , Šmit, Z. , & Tomić, S. (2008). The content of essential and toxic elements in Croatian propolis samples and their tinctures. Journal of Apicultural Research, 47, 35–45.

[fsn33866-bib-0017] Daleprane, J. B. , & Abdalla, D. S. (2013). Emerging roles of propolis: Antioxidant, cardioprotective, and antiangiogenic actions. Evidence‐Based Complementary and Alternative Medicine, 2013, 1–8. 10.1155/2013/175135 PMC3638596 23662115

[fsn33866-bib-0018] Dogan, M. , Silici, S. , Saraymen, R. , & Ilhan, I. O. (2006). Element content of propolis from different regions of Turkey. Acta Alimentaria, 35, 127–130.

[fsn33866-bib-0019] Golubkina, N. A. , Sheshnitsan, S. S. , Kapitalchuk, M. V. , & Erdenotsogt, E. (2016). Variations of chemical element composition of bee and beekeeping products in different taxons of the biosphere. Ecological Indicators, 66, 452–457.

[fsn33866-bib-0020] Gonzalez‐Martin, M. I. , Revilla, I. , Betances‐Salcedo, E. V. , & Vivar‐Quintana, A. M. (2018). Pesticide residues and heavy metals in commercially processed propolis. Microchemical Journal, 143, 423–429.

[fsn33866-bib-0021] Huang, S. , Zhang, C.‐P. , Wang, K. , Li, G. , & Hu, F. L. (2014). Recent advances in the chemical composition of propolis. Molecules, 19, 19610–19632. 25432012 10.3390/molecules191219610 PMC6271758

[fsn33866-bib-0022] Ippolito, E. , Floreno, B. , Rinaldi, C. G. , Trodella, L. , Meroni, F. L. , Iurato, A. , D'Angelillo, R. M. , Ramella, S. , & Fiore, M. (2018). Efficacy of a propolis‐based syrup (FARINGEL) in preventing radiation‐induced esophagitis in locally advanced lung cancer. Chemotherapy, 63, 76–82. 29554652 10.1159/000487897

[fsn33866-bib-0023] Jabot, C. , Fieu, M. , Giroud, B. , Buleté, A. , Casabianca, H. , & Vulliet, E. (2015). Trace‐level determination of pyrethroid, neonicotinoid and carboxamide pesticides in beeswax using dispersive solid‐phase extraction followed by ultra‐high‐performance liquid chromatography‐tandem mass spectrometry. International Journal of Environmental Analytical Chemistry, 95, 240–257.

[fsn33866-bib-0024] Juliano, C. , Pala, C. L. , & Cossu, M. (2007). Preparation and characterisation of polymeric films containing propolis. Journal of Drug Delivery Science and Technology, 17, 177–182.

[fsn33866-bib-0025] Kieliszek, M. , Piwowarek, K. , Kot, A. M. , Wojtczuk, M. , Roszko, M. , Bryła, M. , & Petkoska, A. T. (2023). Recent advances and opportunities related to the use of bee products in food processing. Food Science & Nutrition, 11, 4372–4397. 37576029 10.1002/fsn3.3411 PMC10420862

[fsn33866-bib-0026] Liao, N. , Sun, L. , Wang, D. , Chen, L. , Wang, J. , Qi, X. , Zhang, H. , Tang, M. , Wu, G. , Chen, J. , & Zhang, R. (2021). Antiviral properties of propolis ethanol extract against norovirus and its application in fresh juices. LWT, 152, 112169.

[fsn33866-bib-0027] Lozano, A. , Hernando, M. D. , Uclés, S. , Hakme, E. , & Fernández‐Alba, A. R. (2019). Identification and measurement of veterinary drug residues in beehive products. Food Chemistry, 274, 61–70. 30372985 10.1016/j.foodchem.2018.08.055

[fsn33866-bib-0028] Luo, Y.‐J. , Yang, Z.‐G. , Xie, D.‐Y. , Ding, W. , da, A. S. , Ni, J. , Chai, J. P. , Huang, P. , Jiang, X. J. , & Li, S. X. (2014). Molecular cloning and expression of glutathione S‐transferases involved in propargite resistance of the carmine spider mite, *Tetranychus cinnabarinus* (Boisduval). Pesticide Biochemistry and Physiology, 114, 44–51. 25175649 10.1016/j.pestbp.2014.07.004

[fsn33866-bib-0029] Matić, P. , Sabljić, M. , & Jakobek, L. (2017). Validation of spectrophotometric methods for the determination of total polyphenol and Total flavonoid content. Journal of AOAC International, 100, 1795–1803. 28730980 10.5740/jaoacint.17-0066

[fsn33866-bib-0030] Mohammadzadeh, S. , Sharriatpanahi, M. , Hamedi, M. , Amanzadeh, Y. , Sadat Ebrahimi, S. E. , & Ostad, S. N. (2007). Antioxidant power of Iranian propolis extract. Food Chemistry, 103, 729–733.

[fsn33866-bib-0031] Morales, M. M. , Ramos, M. J. G. , Vázquez, P. P. , Díaz Galiano, F. J. , García Valverde, M. , Gámiz López, V. , Manuel Flores, J. , & Fernández‐Alba, A. R. (2020). Distribution of chemical residues in the beehive compartments and their transfer to the honeybee brood. Science of the Total Environment, 710, 136288. 31927284 10.1016/j.scitotenv.2019.136288

[fsn33866-bib-0032] Oruç, H. H. , Sorucu, A. , Ünal, H. H. , & Aydin, L. (2017). Effects of season and altitude on biological active certain phenolic compounds levels and partial standardization of propolis. Ankara Üniversitesi Veteriner Fakültesi Dergisi, 64, 13–20.

[fsn33866-bib-0033] Ozdal, T. , Ceylan, F. D. , Eroglu, N. , Kaplan, M. , Olgun, E. O. , & Capanoglu, E. (2019). Investigation of antioxidant capacity, bioaccessibility and LC‐MS/MS phenolic profile of Turkish propolis. Food Research International, 122, 528–536. 31229108 10.1016/j.foodres.2019.05.028

[fsn33866-bib-0034] Ozdal, T. , Sari‐Kaplan, G. , Mutlu‐Altundag, E. , Boyacioglu, D. , & Capanoglu, E. (2018). Evaluation of Turkish propolis for its chemical composition, antioxidant capacity, anti‐proliferative effect on several human breast cancer cell lines and proliferative effect on fibroblasts and mouse mesenchymal stem cell line. Journal of Apicultural Research, 57, 627–638.

[fsn33866-bib-0035] Papotti, G. , Bertelli, D. , Bortolotti, L. , & Plessi, M. (2012). Chemical and functional characterization of Italian propolis obtained by different harvesting methods. Journal of Agricultural and Food Chemistry, 60, 2852–2862. 22360702 10.1021/jf205179d

[fsn33866-bib-0036] Pellati, F. , Orlandini, G. , Pinetti, D. , & Benvenuti, S. (2011). HPLC‐DAD and HPLC‐ESI‐MS/MS methods for metabolite profiling of propolis extracts. Journal of Pharmaceutical and Biomedical Analysis, 55, 934–948. 21497475 10.1016/j.jpba.2011.03.024

[fsn33866-bib-0037] Pérez‐Parada, A. , Colazzo, M. , Besil, N. , Geis‐Asteggiante, L. , Rey, F. , & Heinzen, H. (2011). Determination of coumaphos, chlorpyrifos and ethion residues in propolis tinctures by matrix solid‐phase dispersion and gas chromatography coupled to flame photometric and mass spectrometric detection. Journal of Chromatography. A, 1218, 5852–5857. 21782188 10.1016/j.chroma.2011.06.097

[fsn33866-bib-0038] Popova, M. P. , Bankova, V. S. , Bogdanov, S. , Tsvetkova, I. , Naydenski, C. , Marcazzan, G. L. , & Sabatini, A. G. (2007). Chemical characteristics of poplar type propolis of different geographic origin. Apidologie, 38, 306–311.

[fsn33866-bib-0039] Pose‐Juan, E. , Rial‐Otero, R. , Martínez‐Carballo, E. , López‐Periago, E. , & Simal‐Gándara, J. (2009). Determination of metalaxyl and identification of adjuvants in wettable powder pesticide technical formulas. Analytical and Bioanalytical Chemistry, 394, 1535–1544. 19194695 10.1007/s00216-009-2633-z

[fsn33866-bib-0040] Ristivojević, P. , Trifković, J. , Andrić, F. , & Milojković‐Opsenica, D. (2015). Poplar‐type propolis: Chemical composition, botanical origin and biological activity. Natural Product Communications, 10, 1934578X1501001117. 26749815

[fsn33866-bib-0041] Roberson, C. E. , & Hem, J. D. (1969). Solubility of aluminum in the presence of hydroxide fluoride, and sulfate. Chemistry of aluminum in natural water. United States Government Printing Office.

[fsn33866-bib-0042] Rufatto, L. C. , Luchtenberg, P. , Garcia, C. , Thomassigny, C. , Bouttier, S. , Henriques, J. A. P. , Roesch‐Ely, M. , Dumas, F. , & Moura, S. (2018). Brazilian red propolis: Chemical composition and antibacterial activity determined using bioguided fractionation. Microbiological Research, 214, 74–82. 30031483 10.1016/j.micres.2018.05.003

[fsn33866-bib-0043] Sales, A. , Alvarez, A. , Areal, M. R. , Maldonado, L. , Marchisio, P. , Rodríguez, M. , & Bedascarrasbure, E. (2006). The effect of different propolis harvest methods on its lead contents determined by ET AAS and UV‐visS. Journal of Hazardous Materials, 137(3), 1352–1356. 16787698 10.1016/j.jhazmat.2006.05.026

[fsn33866-bib-0044] Silici, S. (2008). Farklı botanik orijine sahip propolis örneklerinde biyolojik olarak aktif bileşiklerin belirlenmesi. Journal of Erciyes Uni Graduate School of Natural and Applied Sciences, 24, 120–128.

[fsn33866-bib-0045] Singleton, V. L. , & Rossi, J. A. (1965). Colorimetry of total phenolics with phosphomolybdic‐phosphotungstic acid reagents. American Journal of Enology and Viticulture, 16, 144–158.

[fsn33866-bib-0046] Soós, Á. , Bódi, É. , Várallyay, S. , Molnár, S. , & Kovács, B. (2019). Mineral content of propolis tinctures in relation to the extraction time and the ethanol content of the extraction solvent. LWT, 111, 719–726.

[fsn33866-bib-0047] Stawiarz, E. , & Dyduch, J. (2014). Zastosowanie produktów pszczelich pochodzenia roślinnego w apiterapii. Episteme, 25, 111–127.

[fsn33866-bib-0048] Svečnjak, L. , Chesson, L. A. , Gallina, A. , Maia, M. , Martinello, M. , Mutinelli, F. , Muz, M. N. , Nunes, F. M. , Saucy, F. , Tipple, B. J. , Wallner, K. , Waś, E. , & Waters, T. A. (2019). Standard methods for *Apis mellifera* beeswax research. Journal of Apicultural Research, 58, 1–108.

[fsn33866-bib-0049] Victorino, F. R. , Bramante, C. M. , Watanabe, E. , Ito, I. Y. , Franco, S. L. , & Hidalgo, M. M. (2009). Antibacterial activity of propolis‐based toothpastes for endodontic treatment. Brazilian Journal of Pharmaceutical Sciences, 45, 795–800.

