# Supplementary material for: Effect of wax separation on macro‐ and micro‐elements, phenolic compounds, pesticide residues, and toxic elements in propolis
Source: Food Sci Nutr. 2023 Dec 6;12(3):1736–48. doi: 10.1002/fsn3.3866 (PMC10916619; doi:10.1002/fsn3.3866)
Supplement: Supplementary file 1 — Table S1 [file FSN3-12-1736-s001.docx]

**Supporting Information**

**Table S1. LC-MS/MS acquisition method parameters**

| **Compound** | **RT**  **(min)** | **Transitions** | **DP (volts)** | **EP**  **(volts)** | **CE**  **(volts)** |
| --- | --- | --- | --- | --- | --- |
| p-coumaric acid | 2.33 | **MRM 1** 162.894>119.0 | -30 | -8.5 | -20 |
|  |  | **MRM 2** 162.894>93.0 | -30 | -8.5 | -38 |
| 3,4 dihydroxybenzoic acid | 2.20 | **MRM 1** 152.891>108.9 | -35 | -4.5 | -22 |
|  |  | **MRM 2** 152.891>108.3 | -35 | -4.5 | -30 |
| quercetin | 2.48 | **MRM 1** 300.833>150.9 | -65 | -9.5 | -30 |
|  |  | **MRM 2** 300.833>179.1 | -65 | -9.5 | -22 |
| caffeic acid | 2.26 | **MRM 1** 178.947>135.0 | -35 | -4.5 | -22 |
|  |  | **MRM 2** 178.947>134.1 | -35 | -4.5 | -30 |
| t-ferulic acid | 2.35 | **MRM 1** 195.007>177.1 | 36 | 9 | 11 |
|  |  | **MRM 2** 195.007>145.2 | 36 | 9 | 27 |
| Chlorogenic acid | 2.21 | **MRM 1** 355.168>163.1 | 56 | 4 | 19 |
|  |  | **MRM 2** 355.168>89.0 | 56 | 4 | 69 |
| gallic acid | 2.13 | **MRM 1** 168.744>125.0 | -35 | -4 | -16 |
|  |  | **MRM 2** 168.744>79.0 | -35 | -4 | -28 |
| epicathechin | 2.24 | **MRM 1** 288.908>123.1 | -55 | -4.5 | -40 |
|  |  | **MRM 2** 288.908>109.1 | -55 | -4.5 | -38 |
| pyracathechol | 2.25 | **MRM 1** 108. 928>91.0 | -55 | -4.5 | -22 |
|  |  | **MRM 2** 108.928>81.1 | -55 | -4.5 | -20 |
| sinapic acid | 2.34 | **MRM 1** 222.903>208.0 | -40 | -4.5 | -22 |
|  |  | **MRM 2** 222.903>148.9 | -40 | -4.5 | -28 |
| vanilic acid | 2.28 | **MRM 1** 166.889>108.1 | -50 | -4.5 | -24 |
|  |  | **MRM 2** 166.889>151.9 | -50 | -4.5 | -22 |
| syringic acid | 2.28 | **MRM 1** 196.930>123.0 | -55 | -4.5 | -28 |
|  |  | **MRM 2** 196.930>182.0 | -55 | -4.5 | -24 |
| phlorizin | 2.30 | **MRM 1** 435.026>273.1 | -80 | -4.5 | -18 |
|  |  | **MRM 2** 435.026>166.9 | -80 | -4.5 | -42 |

**Table S2. LC-MS/MS acquisition method parameters**

| **Compound** | **RT**  **(min)** | **Transitions** | **Frag (Volts)** | **CE**  **(volts)** |
| --- | --- | --- | --- | --- |
| Carbendazim | 4.14 | **MRM 1** 192.1>160.1 | 100 | 15 |
|  |  | **MRM 2** 192.1>132.1 | 100 | 33 |
| Thiamethoxam | 4.82 | **MRM 1** 292.1>211.1 | 80 | 5 |
|  |  | **MRM 2** 292.1>181.0 | 80 | 19 |
| Acetamiprid | 5.45 | **MRM 1** 223.1>126.1 | 100 | 17 |
|  |  | **MRM 2** 223.1>56.2 | 100 | 11 |
| Tebuthiuron | 5.85 | **MRM 1** 229.2>172.1 | 110 | 13 |
|  |  | **MRM 2** 229.1>116.1 | 110 | 25 |
| Secbumeton | 6.03 | **MRM 1** 226.3>170.0 | 90 | 16 |
|  |  | **MRM 2** 226.3>141.9 | 90 | 24 |
| Thiophonate Methyl | 6.28 | **MRM 1** 343.0>311.0 | 60 | 2 |
|  |  | **MRM 2** 343.0>151.0 | 60 | 14 |
| Metalaxyl | 6.70 | **MRM 1** 280.2>220.2 | 90 | 7 |
|  |  | **MRM 2** 280.2>192.1 | 90 | 13 |
| Fenproprimorph | 6.83 | **MRM 1** 304.4>147.1 | 150 | 29 |
|  |  | **MRM 2** 304.4>98.2 | 150 | 29 |
| Spiroxamine | 6.84 | **MRM 1** 298.3>144.1 | 120 | 15 |
|  |  | **MRM 2** 298.3>100.1 | 120 | 31 |
| Azoxystrobin | 7.42 | **MRM 1** 404.2>372.2 | 100 | 9 |
|  |  | **MRM 2** 404.2>344.2 | 100 | 23 |
| Tebuconazole | 7.59 | **MRM 1** 308.1>124.9 | 100 | 36 |
|  |  | **MRM 2** 308.1>70.0 | 100 | 16 |
| Trifloxystrobin | 8.52 | **MRM 1** 409.3>206.0 | 100 | 9 |
|  |  | **MRM 2** 409.3>186.0 | 100 | 13 |
| Hexythiazox | 9.02 | **MRM 1** 353.1>271.0 | 100 | 6 |
|  |  | **MRM 2** 353.1>228.0 | 100 | 8 |
| Propargite | 9.05 | **MRM 1** 368.1>231.1 | 90 | 2 |
|  |  | **MRM 2** 368.1>175.0 | 90 | 8 |
